# Cretaceous lacewing larvae with binocular vision demonstrate the convergent evolution of sophisticated simple eyes

**DOI:** 10.1111/1744-7917.13509

**Published:** 2025-02-18

**Authors:** Carolin Haug, Florian Braig, Simon J. Linhart, Derek E. G. Briggs, Roland R. Melzer, Alejandro Caballero, Yanzhe Fu, Gideon T. Haug, Marie K. Hörnig, Joachim T. Haug

**Affiliations:** ^1^ Faculty of Biology Ludwig‐Maximilians‐Universität München (LMU Munich) Planegg‐Martinsried Germany; ^2^ GeoBio‐Center at LMU München Germany; ^3^ Department of Earth and Planetary Sciences Yale University and Yale Peabody Museum New Haven Connecticut USA; ^4^ Bavarian State Collection for Zoology, Bavarian Natural History Collections München Germany; ^5^ Medical Biology and Electron Microscopy Center University Medical Center Rostock Rostock Germany

**Keywords:** Burmese amber, Cretaceous, lacewing larvae, Myanmar amber, Neuroptera, stemmata

## Abstract

Many insects and their relatives are renowned for sophisticated compound eyes, which are also preserved in the fossil record. Yet there are other types of eyes, notably the so‐called stemmata of holometabolans, such as beetles, bees, and butterflies. Stemmata are not as effective as compound eyes, except in some predatory larvae. Here we report three lacewing larvae with large forward‐directed stemmata from Cretaceous Kachin amber, Myanmar. The stemmata are large relative to those of other fossil lacewing larvae, comparable to the simple eyes of modern larvae capable of image formation. The head is very wide in one larva, representing a new type of morphology as demonstrated by a quantitative comparison of the head and stylets of over 400 fossil and extant lacewing larvae. The arrangement of the exceptionally large stemmata of the larvae reported here provides stereoscopic vision. These new specimens demonstrate the convergent evolution of highly developed simple eyes in at least two additional lineages of lacewings, showcasing the enormous diversity of lacewing larvae in the Cretaceous.

## Graphical abstract

We report three ca. 100 million‐year‐old lacewing larvae with extraordinarily large stemmata. One of them additionally has a very wide head, which represents a previously unknown morphology. The arrangement of the stemmata indicates stereoscopic vision in these predatory larvae.

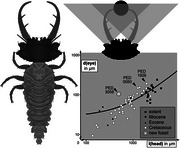

## Introduction

**Fig. 1:**
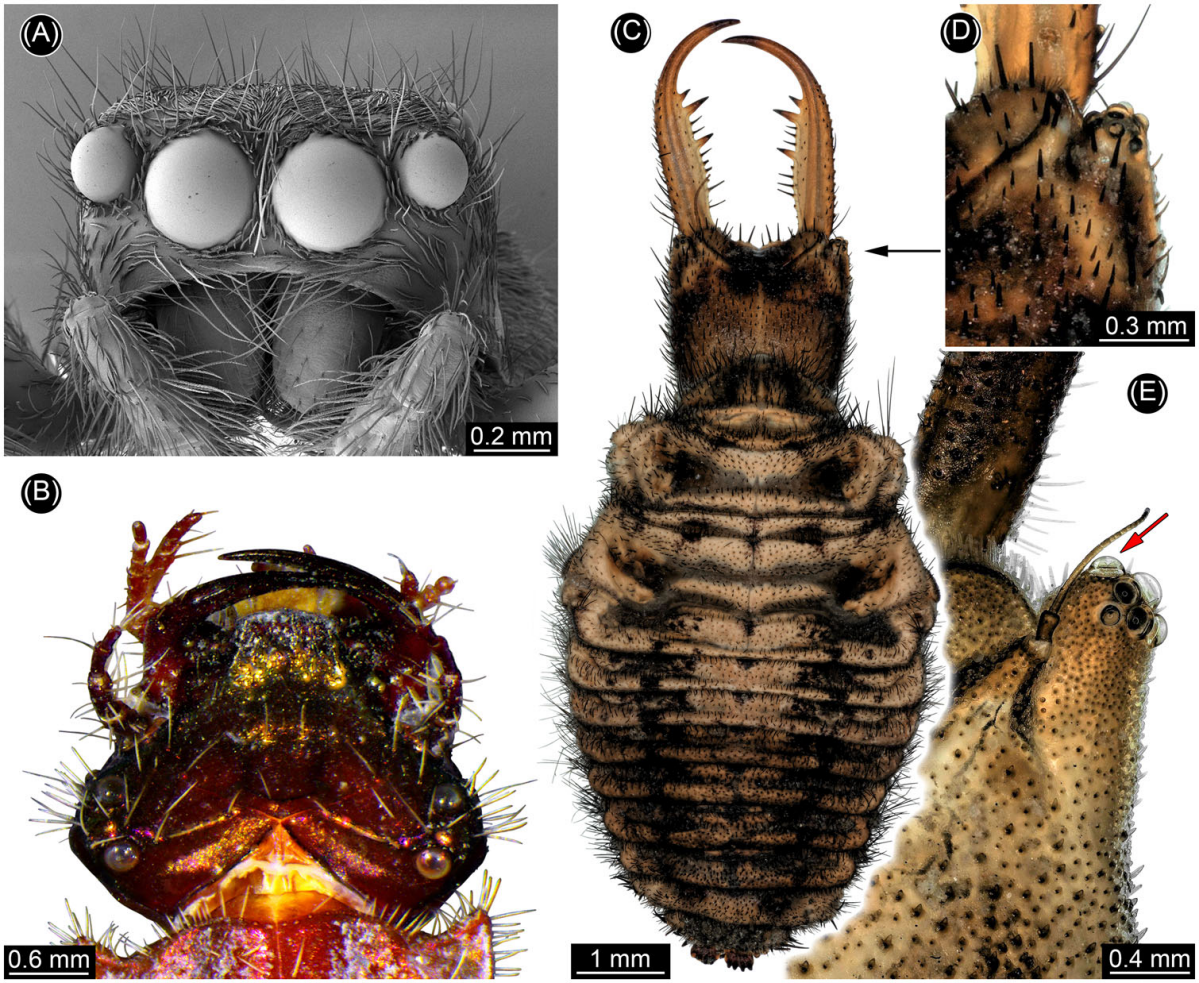
Examples of prominent, sophisticated simple eyes in extant representatives. (A) Jumping spider (scanning electron microscopic image). (B) Tiger beetle larva in dorsal view with prominent stemmata (ZMH 62871). (C, D) Antlion larva (ZSM internal #2). (C) Dorsal view. (D) Eyes. (E) Antlion larva (ZSM internal #12b), close‐up of eyes; note forward facing stemma (arrow).

Reconstructing the morphology and properties of the visual apparatus in fossil organisms offers unique insights into the ecology and behaviour of extinct animals. This approach has been applied to dinosaurs (Lautenschlager *et al.*, 2024), for example, but particularly to the compound eyes of Palaeozoic arthropods including radiodontans (Strausfeld *et al.*, 2016; Paterson *et al.*, 2020), Cambrian arthropods (Lee *et al.*, 2011; Zhao *et al.*, 2013), trilobites (Schoenemann & Clarkson, 2015; Schoenemann, 2021), eurypterids (McCoy *et al.*, 2015; Schoenemann *et al.*, 2019), and crustaceans (Castellani *et al.*, 2012; Schoenemann *et al.*, 2012, 2014; Parker *et al.*, 2013). The eyes of Mesozoic and younger fossil crustaceans have also been documented (Tanaka *et al.*, 2009a; Audo *et al.*, 2016, 2019; Vannier *et al.*, 2016; Jenkins *et al.*, 2022) including those of fossil insects (Parker *et al.*, 1998; Tanaka *et al.*, 2009b; Lindgren *et al.*, 2019; Taylor *et al.*, 2020). Compound eyes are iconic arthropod structures, but many crabs, spiders, and insects have so‐called simple eyes instead of or in addition to compound eyes. The large simple eyes of extant jumping spiders (Fig. 1A), for example, have received considerable attention (Land, 1985; Winsor *et al.*, 2024). Simple eyes have been the subject of little research in fossil arthropods, however, except for those of Cambrian lobopodians (Schoenemann *et al.*, 2009).

The adults and pupae of holometabolan insects—beetles, bees, flies, butterflies, and their close relatives—have compound eyes, which are also present in a few larvae (Melzer, 1994, 2009; Melzer *et al.*, 1994; Saltin, 2015; Saltin *et al.*, 2016). In contrast, the larvae of most holometabolans have a small group of up to seven simple eyes (Paulus, 1986), known as stemmata, on each side of the head. Stemmata are unique to holometabolans, usually simple in structure (Gilbert, 1994), and are often oriented somewhat radially, yielding a wide field of view. The visual fields of right and left stemmata rarely overlap, however, denying the larva binocular vision. Furthermore, most stemmata lack a complex inner structure necessary for image formation. Some predatory holometabolan larvae, in contrast, have evolved enlarged forwardly directed stemmata with overlapping fields of view facilitating binocular vision (Buschbeck, 2014). Examples include larvae of diving beetles, known as water tigers (Mandapaka *et al.*, 2006; Buschbeck *et al.*, 2007), tiger beetles (Toh & Mizutani, 1994; Toh & Okamura, 2007) (Fig. 1B), antlions (Paulus, 1986) (Fig. 1C–E), and whirligig beetles (Lin & Strausfeld, 2013). Stemmata have been reported in more than 120 fossil larvae (Table S1, Text S1), but in no case have image‐forming eyes capable of binocular vision been identified. Here we report larvae of fossil lacewings, neuropterans related to antlions, preserved in Cretaceous amber. The unusually large, forward‐directed stemmata in these predatory larvae are previously unknown and reveal important details of the evolution of highly sophisticated simple eyes in this group.

## Material and methods

### Material

Three specimens from Cretaceous Kachin amber, Myanmar, about 100 million years old (Cruickshank & Ko, 2003; Shi *et al.*, 2012; Yu *et al.*, 2019), were investigated: a new specimen (PED 3058a) and two specimens (PED 0060 and PED 1928) previously figured but without analysis of the eyes (Haug *et al.*, 2020a; Hassenbach *et al.*, 2023). All amber pieces were legally purchased on the platform eBay.com from the traders cretaceous‐burmite and burmite‐miner. They are stored in the Palaeo‐Evo‐Devo Research Group Collection of Arthropods at the Ludwig‐Maximilians‐Universität München. Issues have been raised about the study of amber from Myanmar, especially Kachin amber (e.g., Dunne *et al.*, 2022) and parachute science. We have considered these concerns in detail (Haug *et al.*, 2020b); our strategy is to increase collaboration with colleagues from Myanmar (Haug *et al.*, 2023a, 2023b).

Extant specimens for comparison came from two collections, the Zoological State Collection Munich (ZSM) and the Centrum für Naturkunde (CeNak), Leibniz‐Institut zur Analyse des Biodiversitätswandels (LIB) Hamburg (formerly the collection of the Zoologisches Museum Hamburg— ZMH). We studied two specimens of antlions (ZSM unnumbered; internal #2 and #12b) as well as a tiger beetle larva (ZMH 62871) and antlion‐like larva (ZMH 62887). All specimens were photographed in their original storage liquid (70% ethanol).

### Methods

The fossil (PED) and extant specimens (ZSM) were imaged on a Keyence VHX‐6000 digital microscope under different light settings as stacks and panorama, processed with the internal software; the HDR function was also used. ZMH specimens were documented with a Canon EOS 650D with a MP‐E 65 mm lens as stacks and panorama. Illumination was provided by a Yongnuo YN24EX E‐TTL twin flash. Light was cross‐polarized. Stacks were fused using CombineZP. Panoramas were stitched using the photomerge function of Adobe Photoshop CS3. The background was removed manually with a lasso tool under high zoom.

Specimen PED 3058a was also documented with X‐ray microcomputed tomography (*µ*CT) using a XRadia MicroXCT‐200 (Carl Zeiss Microscopy GmbH, Jena, Germany). Scans were performed using a 4× objective with X‐ray source settings of 40 kV, 8 W, 4 s exposure time and binning 2 generating images with 1014×1014 px with a system‐based calculated pixel size of 2.03 *µ*m and with a 10× objective, X‐ray source settings 40 kV, 8 W, 4 s exposure time, binning 2 generating images with 1005×1005 px, 1.37 *µ*m pixel size. Tomographic images were reconstructed using XMReconstructor (Carl Zeiss Microscopy GmbH, Jena, Germany), resulting in image stacks (TIFF format). However, the overall X‐ray contrast of the larva in PED 3058a was low and the captured images contained several artifacts, resulting in an uninformative visualization as a volume and surface rendering.

Head shapes of 404 specimens of neuropteran larvae, both extant and fossil, were reconstructed with Inkscape or Adobe Illustrator CS2 using high‐resolution images. The shapes were analyzed using elliptical Fourier analysis (EFA) (Braig *et al.*, 2024) using the Momocs package, version 1.3.2 (Bonhomme *et al.*, 2014) in the R‐statistics environment, version 4.1.0 (R Core Team, 2021). The code used is provided in the Supporting Information. EFA allowed us to quantify the geometrical information of a complex two‐dimensional shape using Fourier transformation to decompose it (Bonhomme *et al.*, 2014; Braig *et al.*, 2024). The shapes were registered with 1459 ± 128 coordinates, centered and scaled (controlling for the influence of differences in scale). We identified 14 harmonics as representing 99% of the variation in the data. The results were analyzed with a principal component analysis (PCA) (Bonhomme *et al.*, 2014; Braig *et al.*, 2024).

## Results

### Detailed description of specimen PED 3058a

**Fig. 2:**
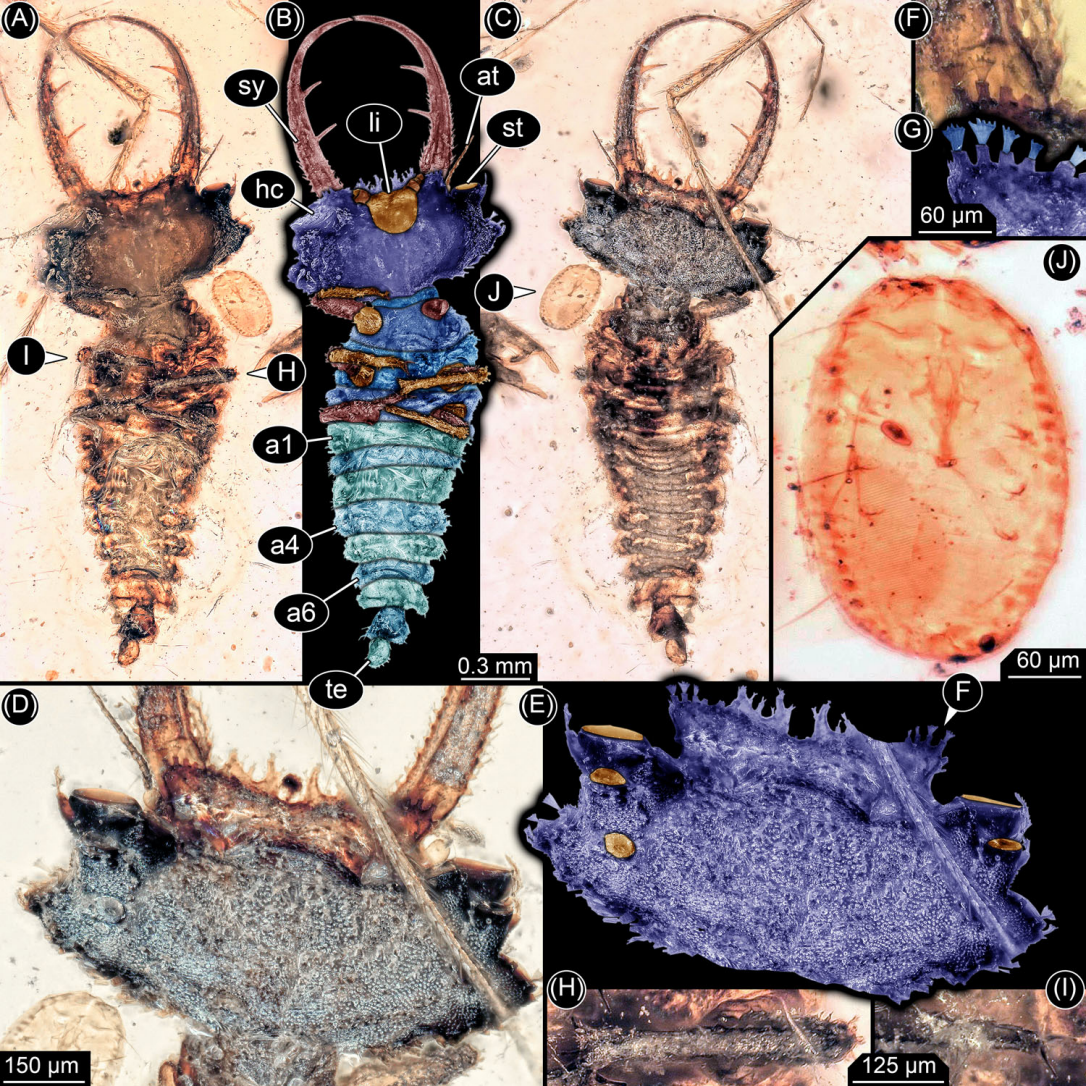
New larva, PED 3058a. (A) Ventral view. (B) Color‐marked version of (A). (C) Dorsal view. (D) Head in dorsal view. (E) Color‐marked version of (D), lenses of stemmata in orange. (F, G) Fringed setae on the head, highlighted in (G). (H) Distal part of leg. (I) Claws. (J) Adjacent whitefly, PED 3058b. Abbreviations: a1–a6 = abdomen segments 1–6; at = antenna; hc = head capsule; li = labium (largely interlocked with head capsule); st = stemma (simple eye); sy = stylet (compound mouthpart formed by mandible and maxilla); te = trunk end.

*General*: Small larva, about 2.7 mm in total length (Fig. 2A–C). Body presumably organized into an ocular segment and 19 postocular segments. Anterior six segments (ocular plus five postocular segments) form capsulate head (Fig. 2D, E). Trunk segmentation clearly defined (Fig. 2A–C). Anterior three trunk segments (thorax) differentiated from more posterior in the possession of a pair of ventral locomotory legs. Posterior trunk (abdomen) differentiated into eight segments and a trunk end which presumably represents several segments.

*Head*: Head capsule rectangular in dorsal view, wider than long, almost 2× (Fig. 2D, E). Anterior rim projecting forward with numerous prominent setae, two larger ones on each side of the mid line flanked by several smaller ones. Setae with robust spine‐like sockets from which four to five short finger‐like features extend (Fig. 2F, G). About five additional setae project from the anterolateral margin of the head capsule with short wide tube‐like sockets, one on the corner of the head capsule (Fig. 2D, E). Anterolateral rim tapers and extends into a triangular protrusion bearing at least two setae with short sockets and more elongate distal features. Posterolateral rim and posterior rim gently curved in general outline, but jagged in detail (Fig. 2D, E). Ocular segment indicated by prominent eyes, at least three stemmata on each side. Largest stemma facing forward on anterolateral socket (Fig. 2D, E). Smaller one lying further posteriorly, directed anterodorsally. Third one even further posteriorly and slightly more medial, directed dorsally.

Postocular segment 1 indicated by antenna which arises posterolaterally from broad anterior protrusion of head capsule. Antenna thin and small, shorter than length of head capsule. Two proximal elements apparent, distal flagellum apparently undivided (Fig. 2A–C). Postocular segment 2 without externally visible structures.

Postocular segments 3 and 4 indicated by mandibles (upper jaws) and maxillae (lower jaws), forming a pair of compound structures called stylets. Stylets about 1.5× length of head capsule, extending forward, tapering with an inward curvature that is most pronounced distally (Fig. 2A–C). Proximal third of each stylet with numerous setae on inner side with distinct sockets and simple distal projections; similar setae on outer lateral margin extend slightly further distally. Two prominent teeth present on inner margin of stylet at mid‐length. Proximal tooth situated at slightly more than one third of length of stylet from its base, distal tooth at slightly less than one third of length of stylet from proximal tooth. Each tooth about as long as proximal width of stylet (Fig. 2A–C). Post‐ocular segment 5 indicated by fused appendages making up a labium (Fig. 2A, B). Proximal part of labium represented by a single sclerite embedded in and functionally connected to head capsule. Anterior margin straight with palp projecting on each side, posterior margin U‐shaped. Palps short and stout with three elements (Fig. 2A, B).

*Trunk*: Region immediately posterior to the head appears unsclerotized (neck region), width less than 30% of head, very short ventrally, slightly longer and triangular dorsally. Postocular segment 6 (prothorax) with a distinct dorsal sclerite, pronotum, V‐shaped opening forward, about 2× width of neck (Fig. 2C). Prothorax with a pair of prominent locomotory legs ventrally. Exact number of elements unclear (Fig. 2A, B). Proximal part (likely coxa and trochanter) short and broad. Next element (femur) more elongate and slender, longer than wide, about 2×. Next (tibia) about as long as femur but narrower (Fig. 2H). Distal element (tarsus) about as wide as tibia, but shorter, with a pair of distal claws (Fig. 2I).

Postocular segment 7 (mesothorax) with a distinct dorsal sclerite, pronotum, slightly V‐shaped, opening anteriorly. Wider but slightly shorter than prothorax with a pair of prominent, locomotory legs, similar to that of prothorax. Postocular segment 8 (metathorax) similar to mesothorax but slightly shorter with a pair of similar legs. Postocular segment 9 (abdomen segment 1) similar in shape to metathorax but lacking appendages. Postocular segments 10–15 (abdomen segments 2–7) similar to anterior segment of abdomen, but progressively decreasing in width (Fig. 2A–C). Postocular segment 16 (abdomen segment 8) widens posteriorly, length similar to maximum width. Trunk end rounded, slightly narrower anteriorly than abdomen segment 8, longer than wide. All trunk segments with a prominent dorsal fold separating an anterior and posterior region. Setae with extended sockets present on lateral margin (Fig. 2A–C).

*Syninclusions*: Small beetle (Coleoptera; not in the image), large fly (Diptera; leg apparent in Fig. 2C), and small hemipteran (Fig. 2J). The small hemipteran may represent a whitefly puparium (Aleyrodidae), based on its extremely small size, general appearance, and diagnostic characteristics, such as the absence of claws on the legs and the lack of dorsal compound pores. This interpretation is consistent with tiny remnants of the legs apparent in the specimen as well as the lack of cuticular structures (Manzari & Fathipour, 2021).

### Shape analysis

The principal component analysis of the head shapes resulted in 23 principal components describing over 99% of variation in the data. PC1, representing 38.1% of the variation, is dominated by the thickness of the tips of the stylets and the protrusions on which the eyes are situated. Negative values represent slim head capsules with thick‐tipped stylets, while positive values represent slim‐tipped stylets with prominent eye protrusions and wider head capsules. PC2, representing 23.2% of the variation, is dominated by the position of the stylets. Negative values represent heads with distally inserting stylets with prominent proximal teeth, whereas positive values represent heads with medially inserting stylets with prominent distal teeth.

**Fig. 3:**
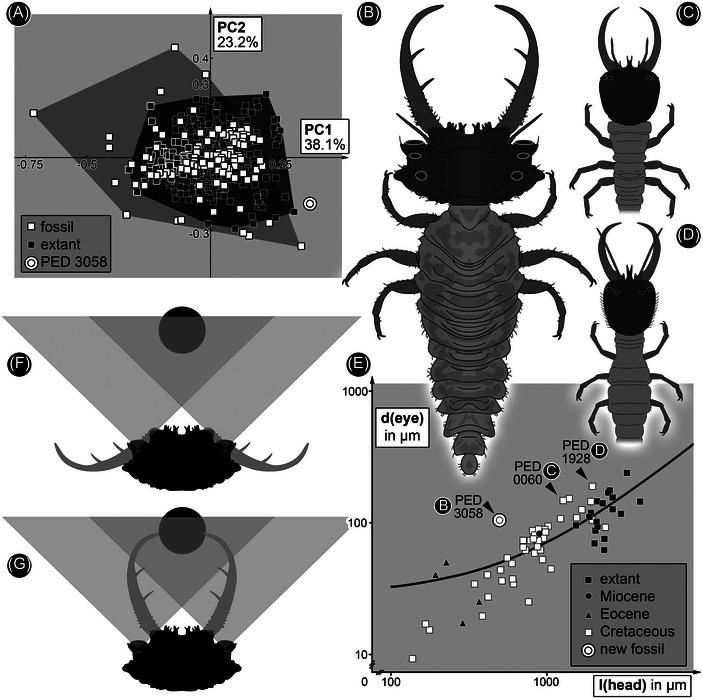
Comparison of antlion‐like larvae. (A) Scatterplot of PC2 vs. PC1 describing the shape of head and stylets of antlion‐like larvae. Fossil larvae occupy a wider area than extant larvae, reflecting earlier results (Braig *et al.*, 2023; Haug *et al.*, 2023c). The new fossil larva plots outside the area of modern larvae and that of known fossils. (B–D) Simplified restorations of fossil larvae with forward‐directed stemmata. (B) PED 3058a. (C) PED 0060. (D) PED 1928. (E) Scatterplot of diameter of the lens of the largest eye vs. length of head of different antlion‐like larvae; the axes are logarithmic; for measurements see Table S2. Arrows mark the three fossil larvae with large forward‐directed lenses. (F, G) Reconstructed field of view of the new larva (in B). (F) Presumed position when waiting for prey (black circle) depicted entering the visual field. (G) Larva closing stylets to capture prey.

The morphospace created by plotting PC2 versus PC1 reveals much overlap between extant and fossil specimens (Fig. 3A). The extant representatives plot in a uniform tightly occupied circle around the center of the morphospace. Fossil representatives plot generally in the same area, but also in a wider area above, below and to the left of the morphospace, indicating slimmer heads with both shorter and longer stylets. The new specimen described here plots on the right of the morphospace, outside the area occupied by extant and fossil representatives, indicating a prominent eye protrusion.

## Discussion

**Fig. 4:**
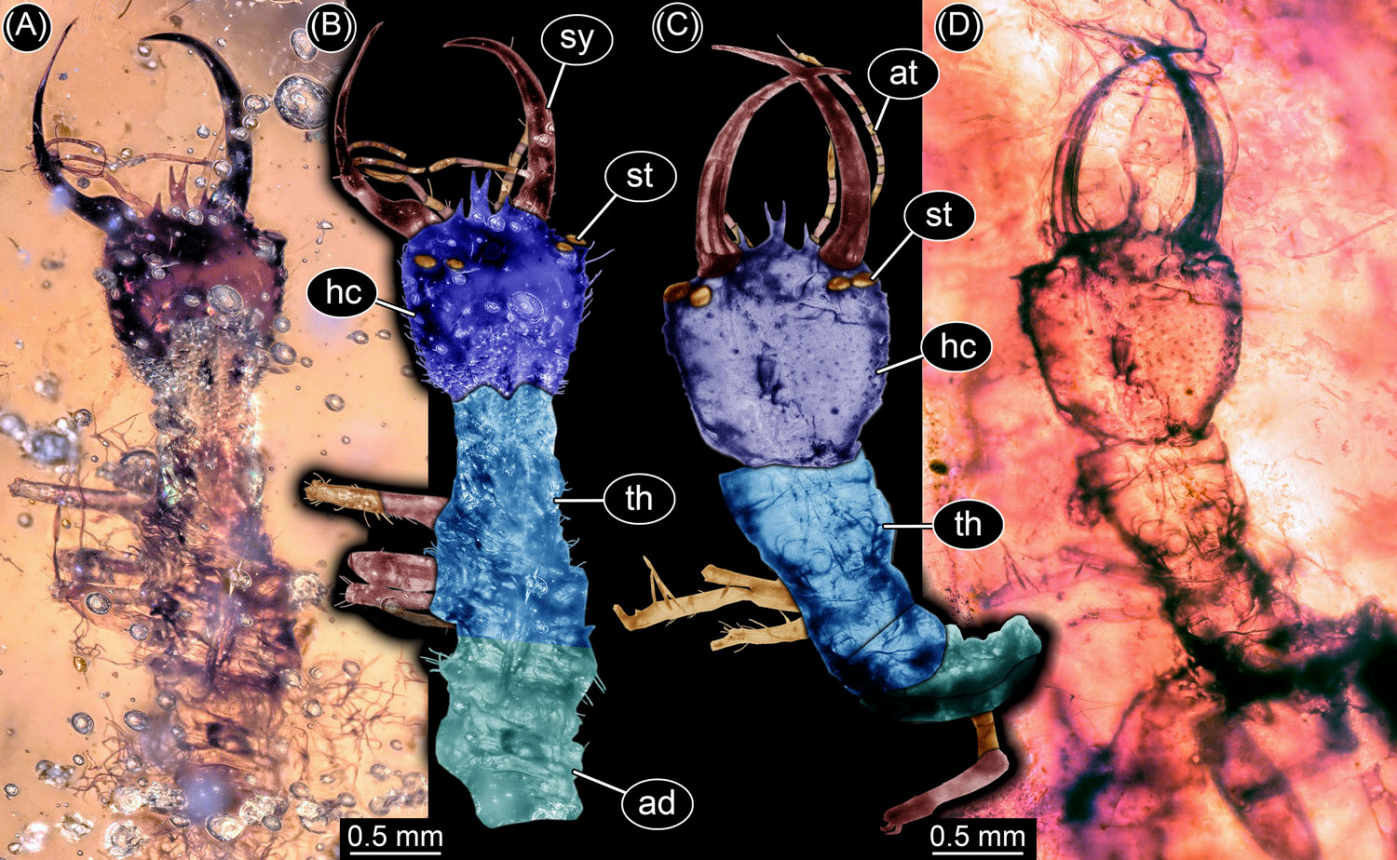
Silky lacewing larvae with forward facing stemmata (from Haug *et al.*, 2020a; Hassenbach *et al.*, 2023). (A, B) PED 0060. (C, D) PED 1928. (A, D) Overview. (B, C) Color‐marked images. Abbreviations: ad = abdomen; at = antenna; hc = head capsule; st = stemma; sy = stylet; th = thorax.

The new specimen of a lacewing larva reported here, PED 3058a, is similar to Cretaceous larvae of a group that includes all the lineages formerly attributed to Ascalaphidae and Myrmeleontidae (Wang *et al.*, 2016). This group is monophyletic, but the name to be assigned to it is currently a matter of debate (Badano *et al.*, 2018, 2021; Jones, 2019; Machado *et al.*, 2019; Haug *et al.*, 2022a). The new specimen differs from all known lacewing larvae. The head (Figs. 2A–E and 3B) is much wider than that of other larvae with similar compound mouthparts (stylets) that likewise bear two teeth (Wang *et al.*, 2016; Badano *et al.*, 2018; Haug *et al.*, 2022a). The width reflects the presence of large forward‐facing eyes. In the analysis of the shape of the head and stylets, the new specimen plots outside all other lacewing larvae (Fig. 3A). The new specimen also lies well above the trend line of a plot of the diameter of the eye versus the length of the head (Fig. 3E). Larger larvae that plot above the trend line are dominated by extant antlions, which have one pair of forward‐directed image‐forming stemmata, but three additional medium‐sized Cretaceous larvae also plot above the line. One is a larva from French amber with laterally facing stemmata (Haug *et al.*, 2022a: fig. 2). The other two are long‐nosed antlions, larvae of silky lacewings, with forward facing stemmata (Figs. 3C and 4D) preserved in Kachin amber.

**Fig. 5:**
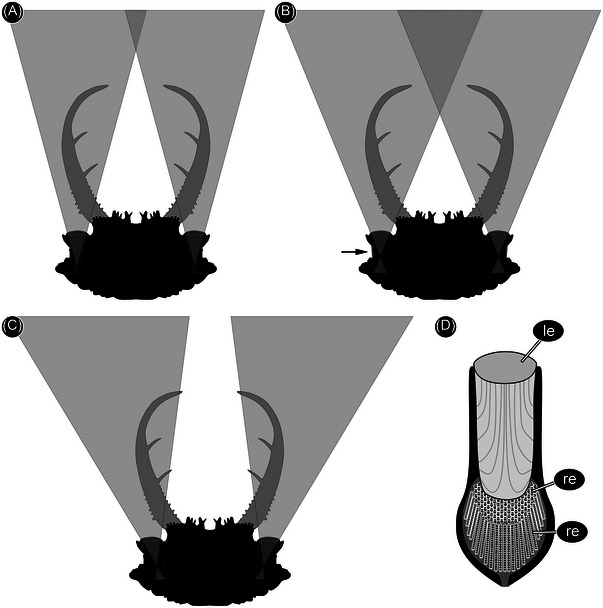
Alternative reconstructions of field of view for the new fossil larva (PED 3058a). (A) Longer inner eye structures are only possible if the eye tapers strongly posteriorly; the resulting area of stereoscopic vision is in front of the stylets. This arrangement is unlikely as such tapering eye shapes are not known for stemmata. (B) Longer eyes without tapering could yield an area of stereoscopic vision between the stylets, but the edge of the eyes is not accommodated within the head capsule (arrow). (C) Longer eyes could also be accommodated if the eyes were not perpendicular to the lens, but this would yield an area of stereoscopic vision far in front of the stylets or none at all. Such an arrangement is also unknown for stemmata. (D) Elongate simple eye of a modern water tiger (simplified from Buschbeck, 2014: fig. 4D, p. 2821). Abbreviations: le = lens; re = retina.

**Fig. 6:**
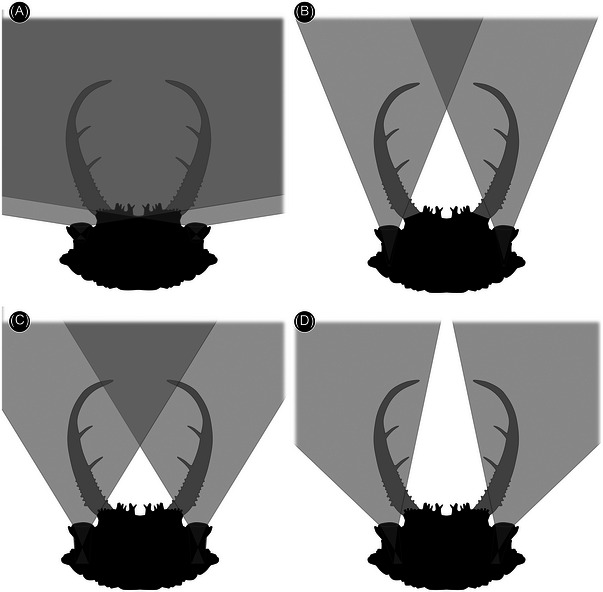
Alternative reconstructions of field of view for the new fossil larva (PED 3058a) assuming a refractive index of 1.4. (A) As in Fig. 3G; a very large part of the field of view is stereoscopic. (B) As in Fig. 5A; here the region of stereoscopic vision is within the range of the stylets. (C) As in Fig. 5B. (D) As in Fig. 5C.

We reconstructed the field of view of the largest stemmata of the new larva (PED 3058a). These are forward facing with the largest diameter, and therefore most important for the ecology of the animal. The reconstruction of the visual field is based on three assumptions. Firstly, the lenses are almost flat on both sides, based on their appearance in the specimens. This is unusual for such stemmata, which often have strongly bulging lenses. It is unlikely that the shape is a result of taphonomic processes. Flat lenses are typical of aquatic larvae, but that would require another dioptric apparatus (Figs. 4 and 5D). Furthermore, all known lacewing larvae with toothed stylets are terrestrial and the fossil shows no evidence of an aquatic lifestyle (gills, swimming hairs). Thus, we reconstructed the field of view based on the flat shape of the eye (Fig. 5) and, as an alternative, with a reasonable refractive index of 1.4 (Berry *et al.*, 2007) (Fig. 6). A *µ*CT scan did not resolve any structures within the eyes, so we assumed, secondly, that the eye and its inner structures are oriented perpendicular to the flat lens (Fig. 5C). Thirdly, we assumed that the dimensions of the eye were constrained by the shape of the head. The head is very wide but short such that maximizing the length of the inner part of the eye would require strong tapering posteriorly (Fig. 5), an unlikely morphology compared to other stemmata. These assumptions demand a short eye with a relatively large opening angle. The possible stereoscopic field of view lies between the stylets. Vision was probably limited in the absence of a strong dioptric apparatus, but the area of higher resolution lay in the area of the stylets.

**Fig. 7:**
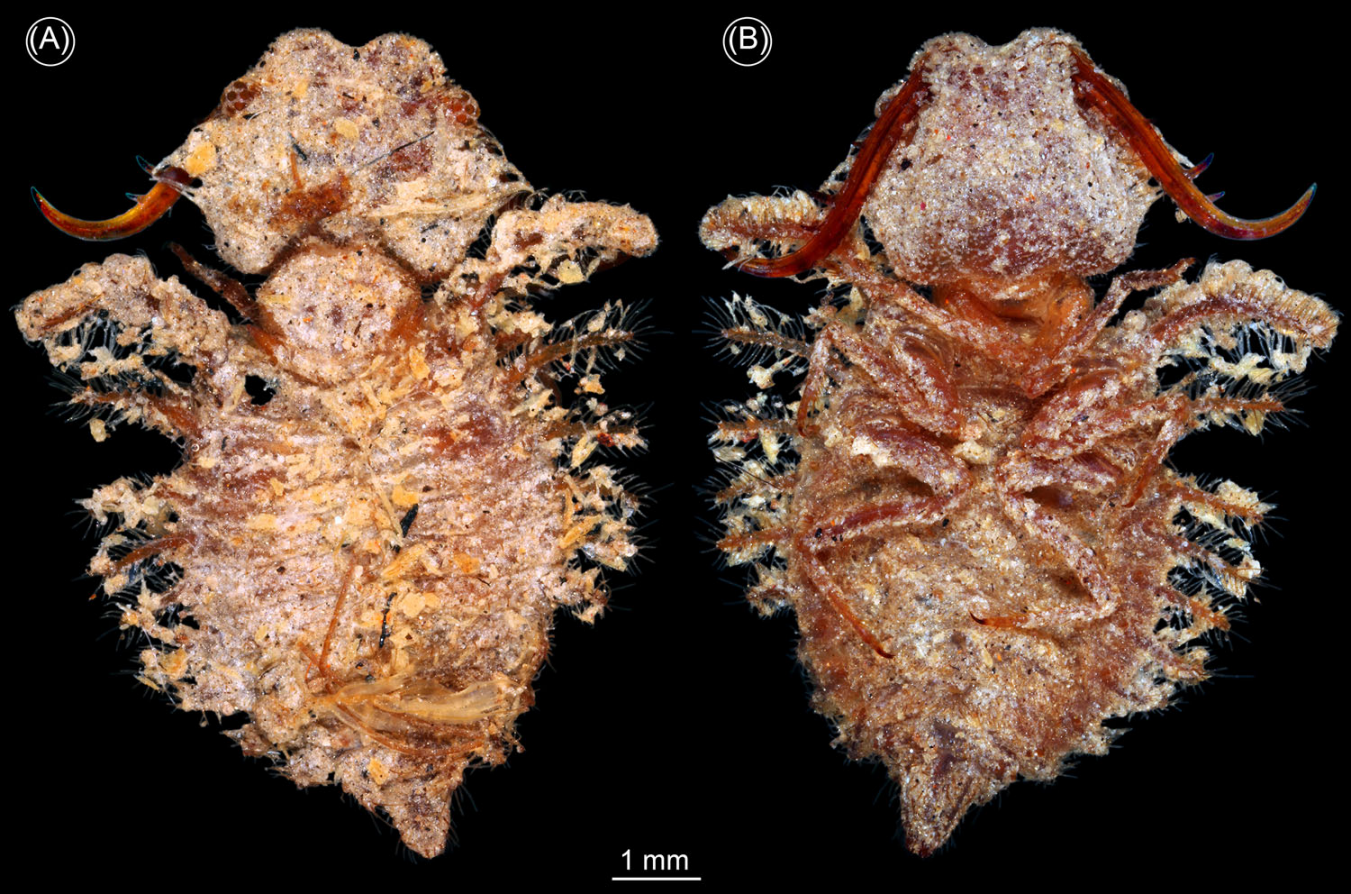
Extant antlion‐like larva (“owllion” larva) with backward‐folded stylets (ZMH 62887). (A) Dorsal view. (B) Ventral view.

The orientation of the eyes in the new specimen, and in the two long‐nosed antlions, indicates that the field of view on the left and right side of the head overlapped (Fig. 3F, G). This applies even without assuming a refractive index of 1.4, and for some of the other possible eye morphologies assuming a refractive index of 1.4 (Fig. 6). An unobstructed view required the stylets to be folded back under the eyes, as in extant relatives of antlions (Figs. 3F and 7) (MacLeod, 1964: figs. 81 and 82). A precise estimate of the field of view of the new specimen is difficult as the shape of the eyes is not well preserved. However, the short head would not accommodate elongate eyes (Fig. 5), as for example in water tigers (Fig. 6D). Hence, it is likely that the eyes were short like those in extant antlions, which would yield wide overlap in the field of view (Fig. 3F). The three larvae investigated here had a visual system similar to that of modern antlions, tiger beetles, and water tigers. However, the flat nature of the lenses resulted in a relatively small field of view. Nonetheless, the eyes would have detected motion of a prey item entering the range of the stylets, which presumably triggered the capture response (as in tiger beetles; Pearson, 1988; Mizutani & Toh, 1998).

The Cretaceous lacewing larvae reported here are the first to provide a basis for functional analysis of stemma vision in a fossil. The highly sophisticated simple eyes of predatory larvae evolved not only in antlions, water tigers, and tiger beetles, but at least a further twice convergently (Badano *et al.*, 2018, 2021; Winterton *et al.*, 2018; McKenna *et al.*, 2019; Vasilikopoulos *et al.*, 2020) among extinct lacewing larvae: in the new larva reported here, and the long‐nosed antlion larvae of silky lacewings (Haug *et al.*, 2020a; Hassenbach *et al.*, 2023). Our results reveal a larger diversity of morphology, ecology, and feeding strategies among Cretaceous lacewing larvae than of those of today.

## Disclosure

The authors declare they have no conflict of interests.

## Supporting information




**Text S1**. References for Table S1.
The Shape data set, the R code and a table with PCA coordinates are available via https://doi.org/10.5281/zenodo.11550932.

**Table S1**. Fossil larvae of Holometabola with preserved stemmata.

**Table S2**. Measurements of head lengths and eye diameters of fossil and extant lacewing larvae. Data set numbers refer to numbers used in Haug *et al.* (2022b, 2022c).
