# Supplementary material for: Cretaceous lacewing larvae with binocular vision demonstrate the convergent evolution of sophisticated simple eyes
Source: Insect Sci. 2025 Feb 18;33(1):441–52. doi: 10.1111/1744-7917.13509 (PMC12905476; doi:10.1111/1744-7917.13509)
Supplement: Supplementary file 1 — Text S1. References for Table S1. The Shape data set, the R code and a table with PCA coordinates are available via https://doi.org/10.5281/zenodo.11550932. [file INS-33-441-s001.docx]

**Supplementary Text S1.** References for Table S1.

1. Haug C, Herrera-Flórez AF, Müller P, Haug JT. 2019 Cretaceous chimera–an unusual 100-million-year old neuropteran larva from the “experimental phase” of insect evolution. *Palaeodiv.* **12**, 1–11. (doi:10.18476/pale.v12.a1)
2. Haug JT, Müller P, Haug C. 2019 A 100-million-year old predator: a fossil neuropteran larva with unusually elongated mouthparts. *Zool. Letts.* **5**, 29. (doi:10.1186/s40851-019-0144-0)
3. Haug JT, Pazinato PG, Haug GT, Haug C. 2020 Yet another unusual new type of lacewing larva preserved in 100-million-year old amber from Myanmar. *Riv. Ital. Paleo. Strati.* **126**, 821–832. (doi:10.13130/2039-4942/14439)
4. Haug GT, Haug C, Pazinato PG, Braig F, Perrichot V, Gröhn C, Müller P, Haug JT. 2020 The decline of silky lacewings and morphological diversity of long-nosed antlion larvae through time. *Palaeo. Electron.* **23(2)**, a39. (doi:10.26879/1029)
5. Haug C, Haug GT, Baranov VA, Solórzano-Kraemer MM, Haug JT. 2021 An owlfly larva preserved in Mexican amber and the Miocene record of lacewing larvae. *Bol. Soc. Geol. Mex.* **73**, A271220.
6. Haug GT, Baranov V, Wizen G, Pazinato PG, Müller P, Haug C, Haug JT. 2021 The morphological diversity of long-necked lacewing larvae (Neuroptera: Myrmeleontiformia). *Bull. Geosci.* **96(4)**, 1–27. (doi:10.3140/bull.geosci.1807)
7. Haug JT, Haug GT, Zippel A, van der Wal S, Müller P, Gröhn C, Wunderlich J, Hoffeins C, Hoffeins H-W, Haug C. 2021 Changes in the morphological diversity of larvae of lance lacewings, mantis lacewings and their closer relatives over 100 million years. *Insects* **12**, 860. (doi:10.3390/insects12100860)
8. Haug JT, Baranov V, Müller P, Haug C. 2021 New extreme morphologies as exemplified by 100 million-year-old lacewing larvae. *Sci. Reps.* **11**, 20432. (doi:10.1038/s41598-021-99480-w)
9. Zippel A, Kiesmüller C, Haug GT, Müller P, Weiterschan T, Haug C, Hörnig MK, Haug JT. 2021 Long-headed predators in Cretaceous amber—fossil findings of an unusual type of lacewing larva. *Palaeoentom*. **004**, 475–498. (doi:10.11646/palaeoentomology.4.5.14)
10. Haug C, Posada Zuluaga V, Zippel A, Braig F, Müller P, Gröhn C, Weiterschan T, Wunderlich J, Haug GT, Haug JT. 2022 The morphological diversity of antlion larvae and their closest relatives over 100 million years. *Insects* **13**, 587. (doi:10.3390/insects13070587)
11. Haug GT, Haug C, van der Wal S, Müller P, Haug JT. 2022 Split-footed lacewings declined over time: indications from the morphological diversity of their antlion-like larvae. *PalZ* **96**, 29–50. (doi:10.1007/s12542-021-00550-1)
12. Haug JT, Linhart S, Haug GT, Gröhn C, Hoffeins C, Hoffeins H-W, Müller P, Weiterschan T, Wunderlich J, Haug C. 2022 The diversity of aphidlion-like larvae over the last 130 million years. *Insects* **13(4)**, 336. (doi:10.3390/insects13040336)
13. Haug JT, van der Wal S, Gröhn C, Hoffeins C, Hoffeins H-W, Haug C. 2022 Diversity and fossil record of larvae of three groups of lacewings with unusual ecology and functional morphology: Ithonidae, Coniopterygidae and Sisyridae. *Palaeo. Electron.* **25**, a14. (doi:10.26879/1212)
14. Hörnig MK, Haug C, Müller P, Haug JT. 2022 Not quite social – possible cases of gregarious behaviour of immatures of various lineages of Insecta preserved in 100-million-year-old amber. *Bull. Geosci.* **97**, 69–87. (doi:10.3140/bull.geosci.1818)
15. Hassenbach C, Buchner L, Haug GT, Haug C, Haug JT. 2023 An expanded view on the morphological diversity of long-nosed antlion larvae further supports a decline of silky lacewings in the past 100 million years. *Insects* **14(2)**, 170. (doi:10.3390/insects14020170)
16. Perrichot V, Engel MS. 2007 Early Cretaceous snakefly larvae in amber from Lebanon, Myanmar, and France (Raphidioptera). *Amer. Mus. Nov.* **3598**, 1–11. (doi:10.1206/0003-0082(2007)3598[1:ECSLIA]2.0.CO;2)
17. Haug C, Haug GT, Zippel A, van der Wal S, Haug JT. 2021 The earliest record of fossil solid-wood-borer larvae—immature beetles in 99 million-year-old Myanmar amber. *Palaeoentom.* **004**, 390–404. (doi:10.11646/palaeoentomology.4.4.14)
18. Liu H, Beutel RG, Makarov KV, Jarzembowski EA, Xiao C, Luo C. 2023 The first larval record of Migadopinae (Coleoptera: Adephaga: Carabidae) from mid-Cretaceous Kachin amber, northern Myanmar. *Cretac. Res.* **142**, 105413. (doi:10.1016/j.cretres.2022.105413)
19. Rosová K, Prokop J, Hammel JU, Beutel RG. 2023 The earliest evidence of Omophroninae (Coleoptera: Carabidae) from mid-Cretaceous Kachin amber and the description of a larva of a new genus. *Arthropod Syst. Phyl.* **81**, 689–704. (doi:10.3897/asp.81.e101374)
20. Zhao X, Zhao X, Jarzembowski EA, Wang B. 2019 The first whirligig beetle larva from mid-Cretaceous Burmese amber (Coleoptera: Adephaga: Gyrinidae). *Cretac. Res.* **99**, 41–45. (doi:10.1016/j.cretres.2019.02.015)
21. Gustafson GT, Michat MC, Balke M. 2020 Burmese amber reveals a new stem lineage of whirligig beetle (Coleoptera: Gyrinidae) based on the larval stage. *Zool. J. Linn. Soc.* **189(4)**, 1232–1248. (doi:10.1093/zoolinnean/zlz161)
22. Engel MS, Grimaldi DA. 2007 The neuropterid fauna of Dominican and Mexican amber (Neuropterida: Megaloptera, Neuroptera). *Amer. Mus. Nov.* **2007(3587)**, 1–58.
23. MacLeod EG. 1970 The Neuroptera of the Baltic Amber. I. Ascalaphidae, Nymphidae, and Psychopsidae. *Psyche* **77(2)**, 147–180.
24. Badano D, Engel MS, Basso A, Wang B, Cerretti P. 2018 Diverse Cretaceous larvae reveal the evolutionary and behavioural history of antlions and lacewings. *Nature Comm.* **9**, 3257. (doi:10.1038/s41467-018-05484-y)
25. Engel MS, Grimaldi DA. 2008 Diverse Neuropterida in Cretaceous amber, with particular reference to the paleofauna of Myanmar (Insecta). *Nova Suppl. Entomol.* **20**, 1–86.
26. Zhang WW. 2017 Frozen Dimensions. The Fossil Insects and Other Invertebrates in Amber. Chongqing: Chongqing University Press, 692 pp.
27. Xia F, Yang G, Zhang Q, Shi G, Wang B. 2015 Amber: Life Through Time and Space. Science Press, Beijing, China, 196 pp.
28. Braig F, Popp T, Zippel A, Haug GT, Linhart S, Müller P, Weiterschan T, Haug JT, Haug C. 2023 The diversity of larvae with multi-toothed stylets from about 100 million years ago illuminates the early diversification of antlion-like lacewings. *Diversity* **15(12)**, 1219. (doi:10.3390/d15121219)
29. Luo C, Liu H, Jarzembowski EA. 2022 High morphological disparity of neuropteran larvae during the Cretaceous revealed by a new large species. *Geol. Mag.* **159(6)**, 954–962. (doi:10.1017/S0016756822000176)
30. Makarkin VN, Wedmann S, Weiterschan T. 2012 First record of a fossil larva of Hemerobiidae (Neuroptera) from Baltic amber. *Zootaxa* **3417(2)**, 53–63. (doi:10.5281/zenodo.281962)
31. Liu X, Zhang W, Winterton SL, Breitkreuz LC, Engel MS. 2016 Early morphological specialization for insect-spider associations in Mesozoic lacewings. *Curr. Biol.* **26**, 1590–1594. (doi:10.1016/j.cub.2016.04.039)
32. Pérez-de la Fuente R, Delclòs X, Peñalver E, Speranza M, Wierzchos J, Ascaso C, Engel MS. 2012 Early evolution and ecology of camouflage in insects. *Proc. Nat. Acad. Scis.* **109(52)**, 21414–21419. (doi:10.1073/pnas.121377511)
33. Pérez-de la Fuente R, Peñalver E, Azar D, Engel MS. 2018 A soil-carrying lacewing larva in Early Cretaceous Lebanese amber. *Sci. Reps.* **8(1)**, 16663. (doi:10.1038/s41598-018-34870-1)
34. Pérez-de la Fuente R, Engel MS, Azar D, Peñalver E. 2019 The hatching mechanism of 130-million-year-old insects: an association of neonates, egg shells and egg bursters in Lebanese amber. *Palaeontology* **62(4)**, 547–559. (doi:10.1111/pala.12414)
35. Pérez-de la Fuente R, Engel MS, Delclòs X, Peñalver E. 2020 Straight-jawed lacewing larvae (Neuroptera) from Lower Cretaceous Spanish amber, with an account on the known amber diversity of neuropterid immatures. *Cretac. Res.* **106**, 104200. (doi:10.1016/j.cretres.2019.104200)
36. Janzen JW. 2002 Arthropods in Baltic Amber. Ampyx Verlag, Halle, 167 pp.
37. Scheven J. 2004 Bernstein-Einschlüsse: Eine untergegangene Welt bezeugt die Schöpfung. Erinnerungen an die Welt vor der Sintflut. Kuratorium Lebendige Vorwelt e.V., Hofheim a.T., 166 pp.
38. Wichard W, Weitschat W. 2004 Im Bernsteinwald. Gerstenberg, Hildesheim. 168 pp.
39. Wichard W, Gröhn C, Seredszus F. 2009 Aquatic insects in Baltic amber. Kessel, Remagen, 336 pp.
40. Kobbert MJ. 2013 Wunderwelt Bernstein: Faszinierende Fossilien in 3-D. WBG, Darmstadt, Germany.
41. Kong B, Shih C, Ren D, Wang Y. 2024 A new giant Jurassic lacewing larva reveals a particular aquatic habit and its significance to the palaeoecology. *J. Syst. Evol.* (doi:10.1111/jse.13071)
42. Haug JT, Baranov V, Schädel M, Müller P, Gröhn C, Haug C. 2020 Challenges for understanding lacewings: how to deal with the incomplete data from extant and fossil larvae of Nevrorthidae? (Neuroptera). *Fragm. entomol.* **52**, 137–167. (doi:10.13133/2284-4880/472)
43. Grimaldi DA. 2000 A diverse fauna of Neuropterodea in amber from the Cretaceous of New Jersey, pp. 259–303. In Grimaldi DA. (ed.), Studies on Fossils in Amber, with Particular Reference to the Cretaceous of New Jersey. Backhuys Publishers, Leiden.
44. Buchner L, Linhart S, Kalmar G, Arce S, Haug GT, Haug JT, Haug C. 2024 New fossil lacewing larvae with trumpet-shaped elongate empodia provide insight into the evolution of this attachment structure. *Riv. Ital. Paleo. Stratigr.* **130**, 67–80. (doi:10.54103/2039-4942/20847)
45. Rasnitsyn AP. 1969 Происхождение и эволюция низших перепончатокрылых. The origin and evolution of lower Hymenoptera [in Russian]. *Trudy Paleontologičeskogo Instituta Akademii Nauk SSSR Moskva* **123**, 1–196.
46. Gröhn C. 2015 Einschlüsse im baltischen Bernstein. Wachholtz Verlag-Murmann Publishers, Kiel, 424 pp.
